# Motion Corrected 3D Whole-Heart Vessel Wall Imaging

**DOI:** 10.1186/1532-429X-18-S1-P323

**Published:** 2016-01-27

**Authors:** Gastao J Lima da Cruz, David Atkinson, Markus Henningsson, Rene M Botnar, Claudia Prieto

**Affiliations:** 1 King's College London, London, United Kingdom; 2 University College London, London, United Kingdom

## Background

Coronary atherosclerosis is not necessarily stenotic, due to outward remodeling of the vessel wall. Plaque burden correlates with risk of coronary disease (Kubo et al, J Am Coll Cardiol 2007) and direct visualization is desired. A 3D flow independent approach for vessel wall imaging was proposed recently (Andia et al, MRM 2013), based on subtraction of data with (T2prep(+)) and without (T2prep(-)) a T2-preparation prepulse. However, T2prep(+) and T2prep(-) data are only accepted when both are within the acquisition window given by the diaphragmatic navigator, leading to increased scan times. Here, we propose to accelerate the acquisition by performing respiratory motion correction on T2prep(-) and T2prep(+) with 100% scan efficiency. This is achieved with a beat-to-beat translational correction combined with a bin-to-bin non-rigid correction.

## Methods

**Figure 1:**
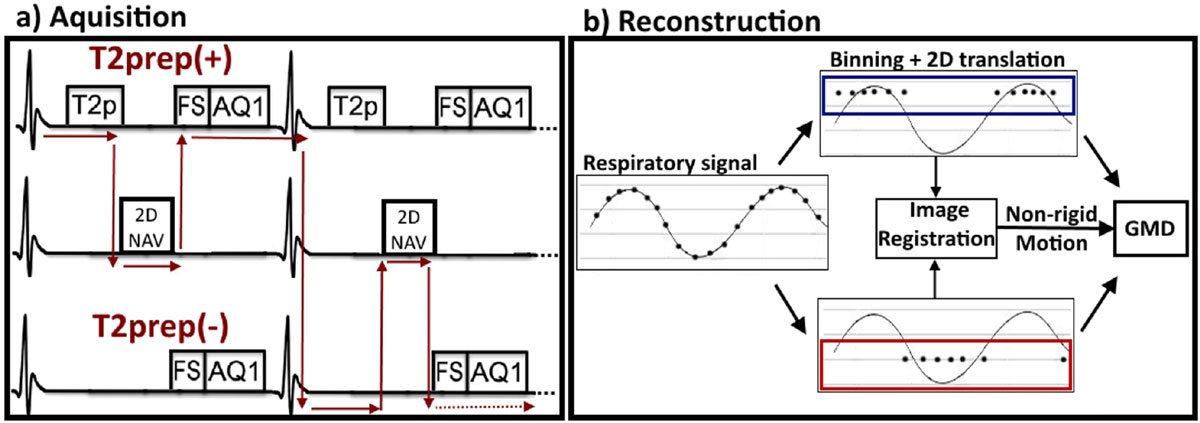
**Proposed interleaved scanning framework: a) T2prep(+) and T2prep(-) data acquisitions are interleaved with a low-resolution 2D image navigator (iNAV)**. These three sequences are setup as independent sequences. b) Foot-head (FH) motion extracted from the iNAV is used to bin data. FH and right-left (RL) motion is corrected within each bin using the iNAV motion estimation. Reconstructed bins from high-resolution data sets are registered to extract non-rigid motion, which is incorporated directly in the final reconstruction.

Data are acquired using an interleaved scanning framework (Henningsson et al, MRM 2014). T2prep(-) and T2prep(+) are acquired interleaved every other heartbeat and preceded by a 2D coronal low resolution image navigator (iNAV) (Figure 1a). Translational foot-head (FH) and right-left (RL) motion is estimated from the iNAV and used to bin data into FH respiratory motion states. Within each bin, 2D translational correction is applied directly in k-space (Figure 1b). Bins are reconstructed with parallel imaging and non-rigid motion is subsequently estimated via image registration. Non-rigid motion between bins is corrected using the General Matrix Description (GMD) (Batchelor et al, MRM 2005).

Eight healthy volunteers were scanned under free-breathing on a 1.5T Philips scanner using a 32-channel coil. Image data was acquired with an ECG-triggered 3D balanced SSFP: coronal slices, flip angle = 70°, TR/TE = 4.6/2.3 ms, FOV = 300 × 300 × 100 mm^3^, resolution = 1 × 1 × 2 mm^3^. iNAV data was acquired with a 2D Golden radial spoiled gradient echo: coronal slice, flip angle = 5°, TR/TE = 2.4/1.1 ms, FOV = 300 × 300 mm^2^, resolution = 4 × 4 mm^2^. Data was reconstructed with no motion correction (NMC), 2D beat-to-beat translation correction (TC) only and with the proposed approach (TC+GMD).

## Results

**Figure 2:**
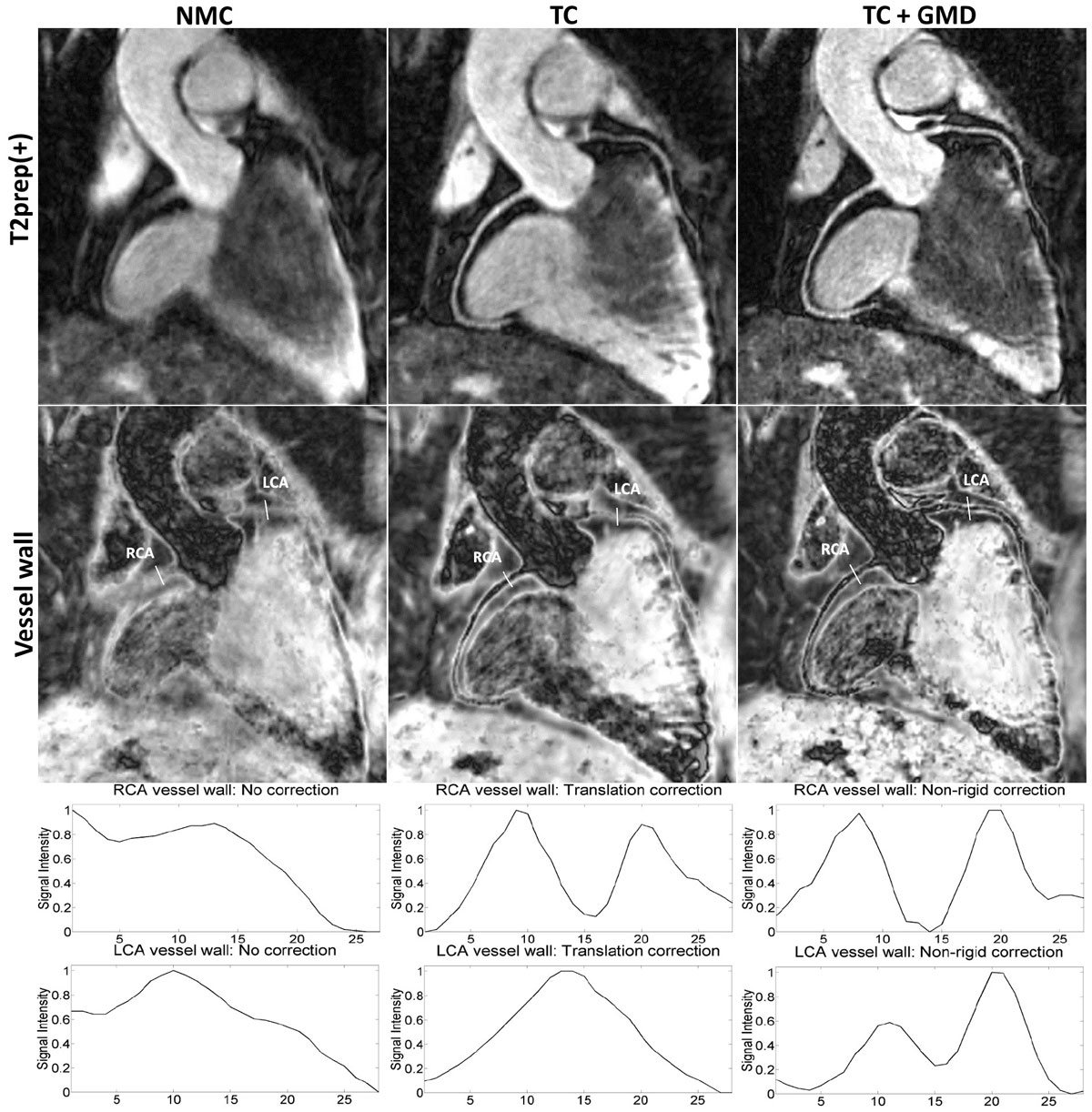
**Top: T2prep images with no motion correction (NMC), 2D translation correction (TC) and proposed approach (TC+GMD)**. Middle: Vessel wall images obtained from T2prep(+) and T2prep(-) with no motion correction (NMC), 2D translation correction (TC) and proposed approach (TC+GMD). Bottom: Normalized signal intensity of vessel wall images along the left and right coronary arteries as depicted in the vessel wall images. Proposed approach leads to clearer delineation of vessel wall in both coronaries.

Reformatted T2prep(+), vessel wall images and intensity profiles along the left and right coronary arteries (LCA and RCA) are shown in Figure 2. Coronary vessels are not visible in NMC due to respiratory motion. A significant improvement is obtained in TC: RCA vessel wall becomes visible, but some residual blurring remains in the LCA. TC+GMD removes further motion artifacts leading to clearer delineation of the vessel wall in both coronary arteries. All volunteers showed visual vessel wall improvement after TC+GMD.

## Conclusions

Good quality vessel wall imaging is achieved with the proposed approach with 100% scan efficiency. The proposed motion correction shows visible improvements over translational correction only, leading to a better delineation of the coronary vessel wall. Future work will focus on additional acceleration using compressed sensing.

